# HKDC1 enhances the proliferation, migration and glycolysis of pancreatic adenocarcinoma and is linked to immune infiltration

**DOI:** 10.7150/jca.92823

**Published:** 2024-02-12

**Authors:** Qiang Pang, Shansong Huang, Jiaqing Cao

**Affiliations:** Department of Gastrointestinal Surgery, The Second Affiliated Hospital of Nanchang University, Nanchang (330006), China.

**Keywords:** Pancreatic adenocarcinoma, Hexokinase Domain Containing 1, prognosis, Tumor microenvironment, Immune infiltration

## Abstract

**Background:** Understanding the molecular mechanisms of pancreatic adenocarcinoma (PAAD) development is vital for treating this disease, as the current prognosis and treatment options are highly discouraging.

**Objective:** This study aimed to examine the involvement of Hexokinase Domain Containing 1 (HKDC1) in the progression of PAAD.

**Methods:** The study utilized bioinformatics techniques to evaluate the relationship between the expression of HKDC1 and clinical characteristics. In vitro experiments were conducted to investigate the molecular mechanisms and biological functions of HKDC1 in PAAD.

**Results:** The findings of this research indicate that the expression of HKDC1 was increased in various types of human cancers, and a significant correlation was observed between elevated HKDC1 expression in PAAD and unfavorable prognosis. According to the findings from univariate and multivariate Cox regression analyses, HKDC1 could potentially serve as a standalone prognostic indicator for individuals diagnosed with PAAD. After performing calculations, we determined that the HKDC1 high-expression group exhibited lower immunologic score and higher ESTIMATE score, indicating a difference in immune infiltration score. In order to validate the expression of HKDC1 in PAAD cell lines, we analyzed the PAAD cell lines through qPCR and protein blotting. The expression of HKDC1 in human PAAD tissues was also detected by western blotting. Additionally, we explored the involvement of HKDC1 in PAAD by conducting experiments such as colony formation, 5-ethynyl-2′-deoxyuridine (EdU), transwell, and wound healing assays. In our study, we discovered that disruption of HKDC1 expression in PAAD cell types resulted in a decrease in cell growth rate and inhibited cell movement and invasion.

**Conclusion:** To conclude, our findings indicate that HKDC1 has a significant impact on the tumor microenvironment (TME) of PAAD and could potentially be a promising target for PAAD treatment, offering fresh perspectives on the management of PAAD.

## Introduction

At present, pancreatic adenocarcinoma (PAAD) is regarded as one of the most lethal cancerous growths, exhibiting an extremely unfavorable prognosis and elevated fatality rate 1. Currently, surgical resection remains the preferred treatment for PAAD. Nevertheless, given the exceedingly hostile characteristics of PAAD, surgical intervention might not provide a definitive cure, and there are instances where surgery is not recommended for certain patients. Despite the application of methods like immunotherapy and targeted therapy, the survival rate of pancreatic cancer remains largely unimproved. Hence, it is crucial to investigate the fundamental processes involved in the progression of PAAD and discover novel treatment targets.

Hexokinase Domain Containing 1 (HKDC1) is a newly discovered protein currently identified as the fifth middle isoform of hexokinase (HK) 2. Previous studies have shown that the HKDC1 gene is located on chromosome 10 and has a high degree of amino acid sequence similarity to HK1^3^, ^4^. In addition, the HKDC1 protein contains a conserved glucose-binding site and an ATP-binding site with the ability to phosphorylate hexose, and Guo et al. demonstrated the HK activity of HKDC1 in vitro and in vivo 3. Taken together, these studies indicate that HKDC1 is a member of the HK family and can participate in glucose metabolism. The fact that HK can perform glucose metabolism by phosphorylating glucose to glucose-6-phosphate, which is the crucial step that limits the rate of glucose metabolism, is widely recognized 5. The remaining four isozymes of HK have been documented in various cancer types and shown to facilitate tumor growth 6-9. Like the other four isozymes, HKDC1 seems to play an important role in mediating glucose metabolism for tumor development. HKDC1 has been reported to promote glycolysis and tumor progression in gastric cancer 10. It has also been shown that HKDC1 promotes tumorigenesis in lung adenocarcinoma by being able to regulate the AMPK/mTOR signaling pathway 11. Moreover, it has additionally been discovered that HKDC1 has the ability to engage with mitochondria and enhance the advancement of hepatocellular carcinoma 12. Nevertheless, the involvement of HKDC1 in PAAD remains unclear.

This study assessed the expression and predictive significance of HKDC1 in PAAD. Furthermore, we explored the correlation between HKDC1 and the tumor microenvironment (TME), as well as immune checkpoints and the responsiveness to drugs. To investigate the function of HKDC1 in PAAD, we conducted various assays including colony formation, EdU, transwell, wound healing, apoptosis, and glucose metabolism.

## Materials and methods

### Obtaining and preparing unprocessed data

Data on HKDC1 expression across multiple cancer types were acquired from gene expression profiling interactive analysis (GEPIA2). To obtain gene expression data (FPKM) and related clinical features, the Cancer Genome Atlas (TCGA) database, was utilized 13. The clinical data used in this study and their correlation with HKDC1 are in the Supplementary Material. Afterwards, we transformed the RNA sequencing data from FPKM to TPM format, kept the clinical data and RNA sequencing data, and assessed all data based on the TCGA release guidelines. Since the data for this inquiry were acquired from the TCGA public repository, there was no need for informed consent or ethical approval. Four postoperative tumour samples from patients with PAAD were collected from the Second Affiliated Hospital of Nanchang University for protein detection. The human specimens used in the study were approved by the Ethics Committee of the Second Affiliated Hospital of Nanchang University.

### Overall survival analysis

The groups were classified into low-expression and high-expression based on the median expression value of HKDC1. Kaplan-Meier analysis and log-rank test were used to visualize the overall survival (OS) curves. To determine whether HKDC1 expression is an independent prognostic factor for PAAD, univariate and multivariate Cox regression analyses were performed.

### Examining the relationship between immune cell infiltration through correlation analysis

The ESTIMATE algorithm 14 was employed to assess the immune score, stromal score, and ESTIMATE score of each sample in the TCGA dataset. This evaluation was conducted on patients categorized into high and low HKDC1 expression groups, and the results were visualized using a violin plot. Furthermore, we utilized gene expression data in CIBERPORT to evaluate the infiltration of immune cells and represented it through histograms. CIBERPORT is a tool for estimating the composition and number of immune cells in mixed cell populations 15. Afterwards, we utilized R software to create bar graphs that illustrate the correlation between different immune cell infiltrations. Furthermore, we examined the association between HKDC1 and various immune checkpoints and displayed the correlation in the form of two heatmaps. Finally, we correlated HKDC1 expression with Tumor burden (TMB).

### Analysis of the sensitivity to drugs and the effectiveness of immunotherapy

In order to determine the sensitivity of PAAD chemotherapeutic drugs to patients with different HKDC1 expression, we used the "pRRophetic" package. PAAD-IPS data were retrieved from TCIA (http://tcia.at/home), a public database for cancer immunomics 16. The immunophenol scores (IPS) were derived from gene expression data, which offered insights into the immunoprofiles of cancer patients undergoing immunotherapy. The data from the immunotherapy were ultimately extracted and visualized using the violin plot based on the treatment regimen.

### Quantitative real-time PCR and western blot

Total RNA was extracted from tissues and cells using Trizol methodology. The cDNA (from TaKaRa, RR047A) was subsequently employed for performing quantitative real-time PCR (qPCR) using TAKARA (RR420A). For the gene HKDC1, the qPCR experiment utilized the following primers: the forward primer with the sequence 5'-ATCCTGGCAAGCAGAGATACG-3' and the reverse primer with the sequence 5'-GACGCTCTGAAATCTGCCCT-3'. GAPDH served as a control for comparison purposes. To analyze whole cell proteins, Western blotting (WB) was conducted with primary antibodies against HKDC1 (1:1000, Proteintech, Wuhan, China) and GAPDH (1:5000, Proteintech, Wuhan, China).

### Assays for staining with 5-ethynyl-2'-deoxyuridine (EdU)

The 100-microliter full medium with 10,000 SW1990 cells was added to 96-well plates and incubated overnight. The SW1990 cell line was established from splenic metastases from a patient with stage II PAAD of the pancreatic exocrine glands and belongs to PAAD. Based on the high expression of HKDC1 in SW1990 and the fact that SW1990 belongs to PAAD, the SW1990 cell line was used for subsequent in vitro studies. Afterwards, the cells were cultured in media with 50 millimolar EdU for a duration of 2 hours. Following fixation using 4% paraformaldehyde, the cells were then exposed to 100 microliters of RiboBIO (Guangzhou, China) for a period of 30 minutes. Afterwards, the cells were rinsed using 0.5% TritonX-100 for a duration of 10 minutes and subsequently subjected to DAPI staining.ImageJ software was used to subsequently determine the quantity of cells that were positive for EdU.

### Colony formation assays

Cells with reduced HKDC1 expression were seeded at a density of 800 cells per well in 6-well plates and cultured at 37°C and 5% CO2 for a duration of two weeks. Afterwards, the cells were treated with 4% paraformaldehyde for 30 minutes, followed by staining with a 1% crystal violet solution for 30 minutes at ambient temperature. Subsequently, the cells were rinsed thrice with PBS. Using an inverted phase contrast microscope, we tallied the count of clones that surpassed 50 cells per colony.

### Cell migration assay

The assessment of cell migration and invasiveness was conducted using chambers that had a pore diameter of 8 millimeters. The cells were placed in a medium without serum and then introduced into the upper compartment (30,000 cells) to conduct migration testing. In the lower section, there was a mixture of 600 microliters of DMEM solution and 10% bovine fetal serum. Following a 24-hour incubation period, the cells were treated with 4% paraformaldehyde for 30 minutes to fix them, and then stained with 1% crystal violet for another 30 minutes. To eliminate any non-migrating cells, a gentle cleaning was performed on the upper surface of the chamber. After fixation and staining, the migration of cells was observed and documented using a phase contrast microscope.

### Wound healing assay

SW1990 cells, which had been transfected with HKDC1 knockdown, were uniformly cultivated in 6-well plates and allowed to grow until reaching complete confluence. Next, the cell layer was delicately scraped using a sterile pipette tip with a volume of 200ul. Cell migration and assessment of the scratched area were examined at the same location under a microscope using ImageJ, both immediately after scratching the cells and after 48 hours.

### Flow cytometry assay

Apoptosis detection was performed using the Cell Double Staining Apoptosis Detection Kit (KGI Bio) which utilizes Annexin-V and propidium iodide (PI). Cell suspensions that had been transfected were centrifuged at room temperature for 5 minutes at a speed of 1,000 rpm. The pellet of cells was suspended again using the buffer for binding. Afterwards, the cells were stained with 5 μl of PI and 5 μl of Annexin V-fluorescein isothiocyanate at room temperature in a dark environment. Flow cytometry was used to analyze apoptosis in the end.

### Determination of glucose and lactate

Using a biochemical analyzer, the glucose and lactate levels in the culture medium were measured after inoculating SW1990 cells with HKDC1 knockdown into 6-well plates.

### Statistical analysis

All statistical analyses were performed using R and GraphPad Prism 9.0.2 software. Mean ± standard deviation was used to express continuous data, and Student's t-test was employed to analyze differences between experimental groups. The Kaplan-Meier method was utilized to generate survival curves, which were then compared using the log-rank test. A significance level of less than 0.05 was deemed statistically significant.

## Results

### HKDC1 is highly expressed in pancreatic cancer tissues and cell lines

**Figure 1:**
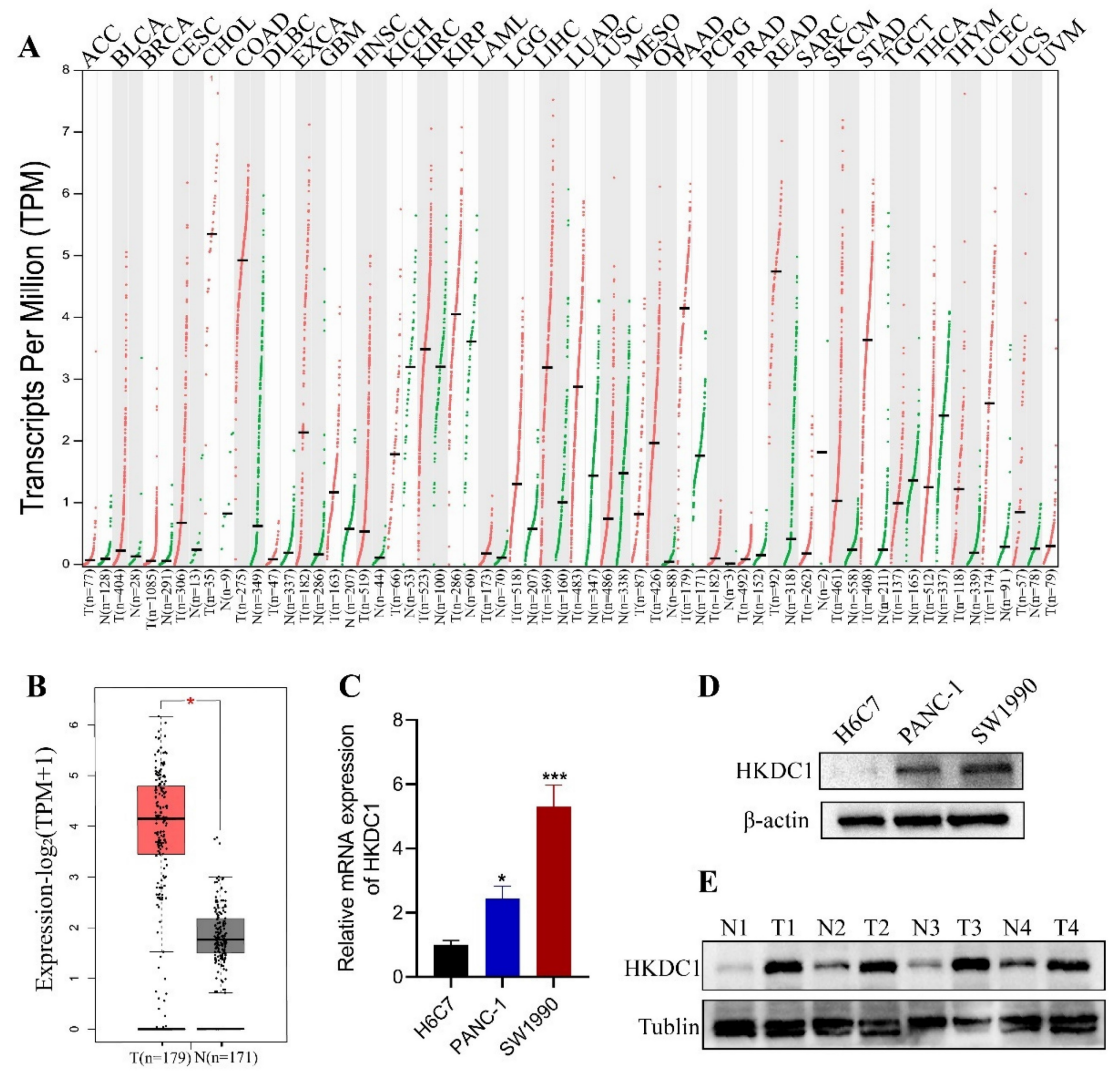
Expression of Hexokinase Domain Containing 1 (HKDC1) in pancreatic adenocarcinoma (PAAD). (A) HKDC1 expression in different malignancies as determined by the GEPIA2 database. (B) The expression of HKDC1 was increased in PAAD tissues compared with adjacent pancreatic tissues. (C) qPCR analysis showed that the expression of HKDC1 mRNA in PAAD cell lines was significantly higher than that in normal pancreatic tissue cell lines. (D) Western blot analysis showed that the expression of HKDC1 protein in PAAD cell line was significantly higher than that in normal pancreatic tissue cell line. (E) Western blotting analysis showed that the expression of HKDC1 protein in human tissue cancer samples was significantly higher than that in paired paracancerous tissues. N: paired paracancerous tissues; T: tumor tissues. * p < 0.05; **p < 0.01; ***p < 0.001; ****p < 0.0001.

**Figure 2:**
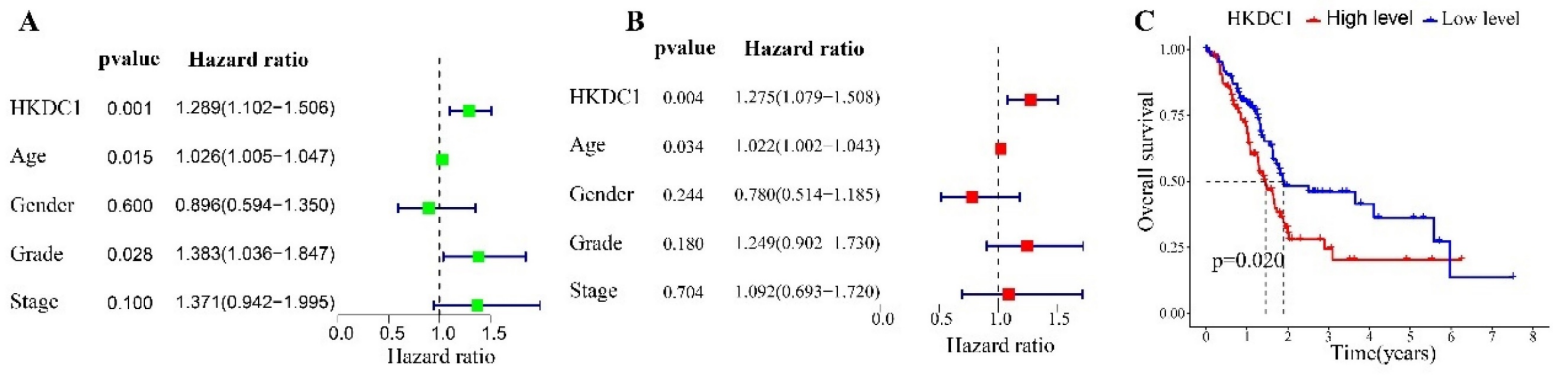
The diagnostic value of HKDC1 expression in PAAD patients. (A) Forest maps of HKDC1 expression and clinical features by univariate Cox regression analysis. (B) Forest maps of HKDC1 expression and clinical features by multivariate Cox regression analysis. (C) Overall survival curves of PAAD patients with different HKDC1 expression levels. * p < 0.05; **p < 0.01; ***p < 0.001; ****p < 0.0001.

Initially, we examined the mRNA expression levels of HKDC1 in different prevalent human malignancies by utilizing the GEPIA2 database. According to the analysis, HKDC1 exhibited high expression levels in various cancer types, such as COAD, ESCA, LIHC, LUAD, OV, PAAD, READ, STAD, THCA, and UCEC tissues. However, there was a tendency of low expression in certain tumors, like KICH and THCA (Figure 1A). In particular, Figure 2A demonstrates the significant overexpression of HKDC1 in tissues affected by pancreatic cancer. Following that, we investigated the expression of HKDC1 in pancreatic tumor cells and normal cells using qPCR and WB (Figure 1C-D). Consistently elevated levels were observed in both HKDC1. HKDC1 showed a clear trend of high expression in SW1990, so SW1990 was mainly used for subsequent studies. In addition, the expression of HKDC1 was detected by testing the tumour samples of PAAD patients and paired paracancerous samples, and HKDC1 was found to be significantly highly expressed in the tumours.

### HKDC1 is an independent predictor of PAAD prognosis

To identify factors linked to unfavorable prognosis in patients with PAAD, we conducted a univariate Cox regression analysis. The examination revealed that older age (HR= 1.026, p = 0.015), higher tumor grade (HR= 1.383, p = 0.028), and increased HKDC1 expression (HR= 1.289, p = 0.001) were strongly linked to unfavorable prognosis (Figure 2A). Following this, we conducted a multiple regression analysis and found that older age (HR= 1.022, p = 0.034) and elevated HKDC1 levels (HR= 1.275, p = 0.004) were significant predictors among PAAD patients (Figure 2B). In order to further examine the predictive importance of HKDC1 expression, we divided PAAD patients into groups based on high and low levels of HKDC1 expression using suitablemcutoffs. The Kaplan-Meier analysis demonstrated that individuals exhibiting elevated levels of HKDC1 expression experienced improved overall survival (OS, p = 0.02) in contrast to those with lower HKDC1 expression (Figure 2C).

### HKDC1 expression is associated with tumor-infiltrating immune cells

**Figure 3:**
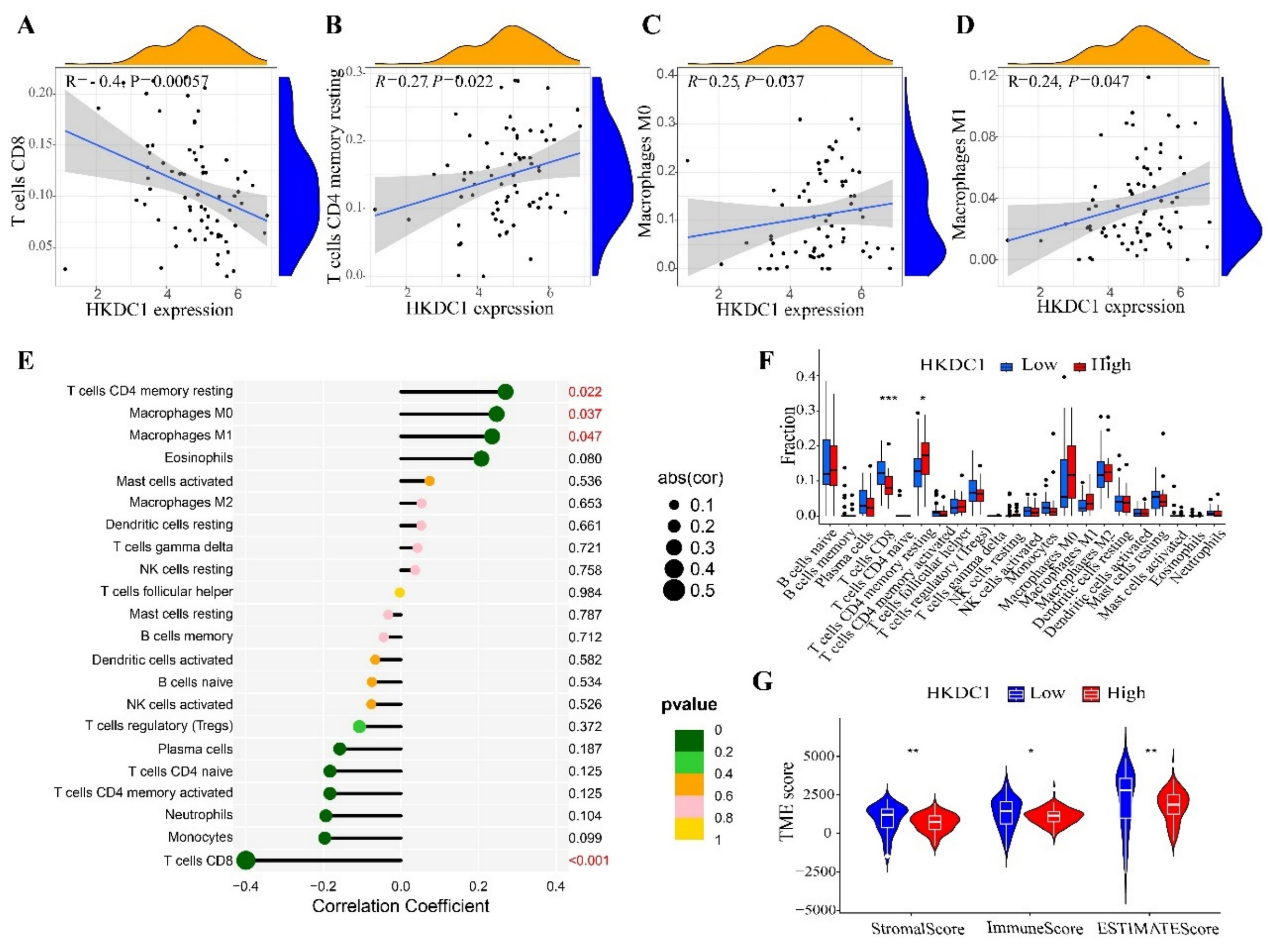
Correlation between HKDC1 expression and tumor microenvironment (TME). (A-D) Scatter plots depict the association between tumor-infiltrating immune cells and HKDC1 expression. (E) Forest map showing the association between HKDC1 expression and 22 tumor-infiltrating immune cells. (F) Differences in the ratio of 22 immune cells to HKDC1 expression levels in PAAD tumor samples. (G) Violin diagram showing the association between TME scores and HKDC1 expression levels. * p < 0.05; **p < 0.01; ***p < 0.001; ****p < 0.0001.

To validate the correlation between HKDC1 expression and immune components in PAAD patients, 22 distinct immune cell profiles were generated using the CIBERSORT technique. The proportion of tumor-infiltrating immune subtypes was then evaluated. The expression of HKDC1 was found to be associated with four distinct categories of immune cells that infiltrate tumors. Among these TICs, T cells CD4 memory resting, Macrophages M0, and Macrophages M1 exhibited a positive correlation with HKDC1 expression, whereas T cells CD8 showed a negative correlation with HKDC1 expression (Figure 3A-F). Furthermore, we computed the immune score, stromal score, and ESTIMATE score for various subcategories, revealing a notable decrease in the immune score, stromal score, and ESTIMATE score within the HKDC1 high-expression cohort (Figure 3G). The data above indicates that HKDC1 might suppress immune reactions in the tumor microenvironment by influencing immune cells. A decrease in the infiltration of immune cells could result in diminished anti-tumor effects, potentially accounting for the unfavorable prognosis observed in patients exhibiting elevated levels of HKDC1 expression.

### High HKDC1 expression may affect immunotherapy

**Figure 4:**
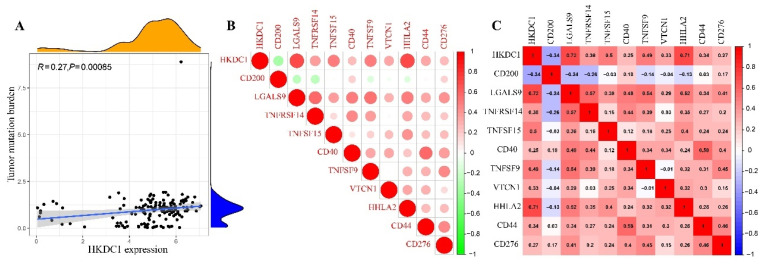
Analysis of HKDC1 expression and tumor mutation burden (TMB) and immunological checkpoint. (A) Correlation between TMB and HKDC1 expression. (B-C) Heat map showing the correlation between HKDC1 and 25 immune checkpoints. The correlation coefficient is shown by the number in each small box. Red indicates a positive correlation between two genes, while blue indicates the opposite. The deeper the shadow, the stronger the association.

**Figure 5:**
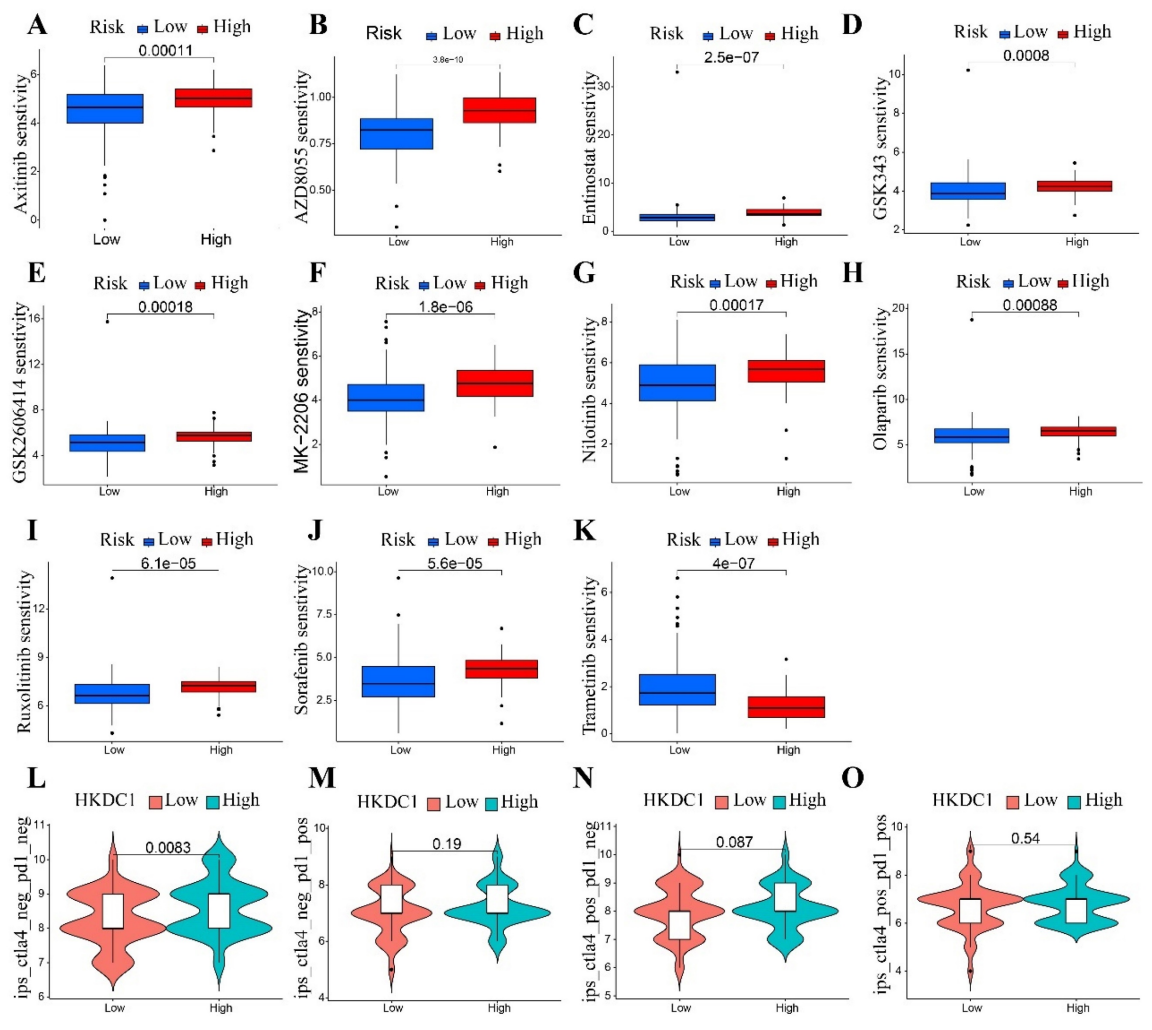
HKDC1 expression as a prognostic factor for tumor immunotherapy. (A-F) Drug sensitivity analysis between two HKDC1 expression subgroups. (G-J) Immunotherapy response between two subgroups expressing HKDC1.

Initially, it was discovered that the expression of HKDC1 showed a positive correlation with the amount of tumors (TMB) (Figure 4A). Furthermore, it was observed that the majority of the immune checkpoints exhibited a positive correlation with the expression of HKDC1, with the exception of CD20. It is worth mentioning that we observed a positive correlation between the expression of HKDC1 and the immune checkpoints LGALS9, TNFRSF14, TNFSF15, CD40, TNFSF9, VTCN1, HHLA2, CD44, and CD2776, as shown in Figure 4B. Afterwards, we assessed the responsiveness of individuals with varying levels of HKDC1 expression in pancreatic cancer to different targeted medications.Increased sensitivity to chemotherapeutic agents like Axitinib, AZD8055, Entinostat, GSK343, GSK2606414, MK-2206, Nilotinib, Olaparib, Ruxolitinib, and Sorafenib was observed in patients exhibiting high HKDC1 expression (Figure 5A-J). Figure 5K showed that patients with high HKDC1 expression had reduced sensitivity to Trametinib compared to other treatments. Ultimately, we assessed the IPS of individuals in various HKDC1 expression categories utilizing the PAAD-IPS cohort. The findings indicated that there was no significant variation in IPS between individuals with elevated and reduced HKDC1 levels (Figure 5L-O).

### HKDC1 promotes pancreatic cancer cell proliferation, migration and invasion and inhibits apoptosis

**Figure 6:**
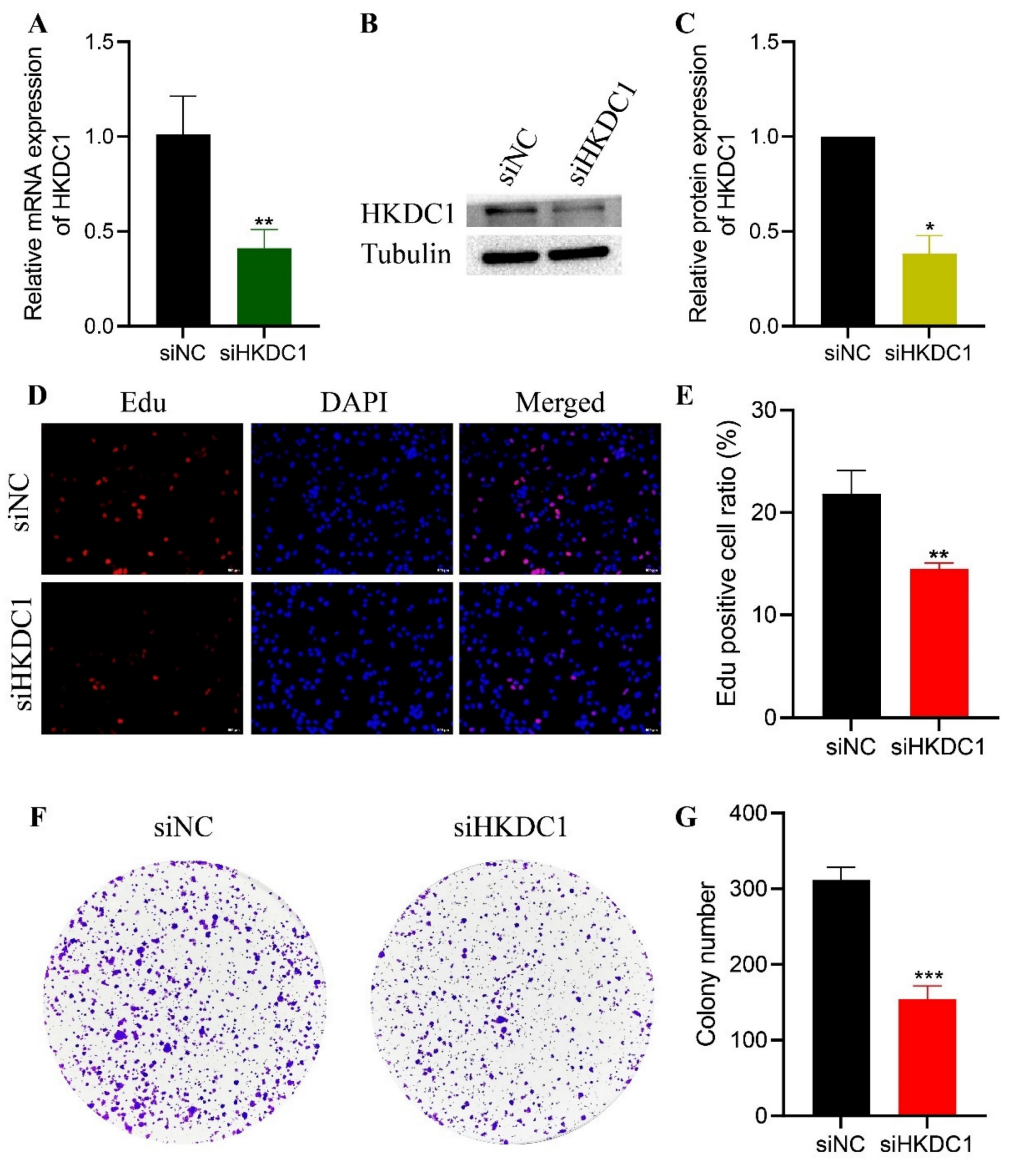
HKDC1 promotes the proliferation of PAAD cells. (A-C) The transfection efficiency of siHKDC1 in SW1990 cells was verified by qPCR and western blot. (D-E) In the siHKDC1 group, the number of SW1990 cells positive for 5-ethynyl-2′-deoxyuridine (EdU) staining was much lower. (F-G) In SW1990 cells, the number of colonies formed in siHKDC1 group was significantly lower than that in siNC group. siNC: knock down interference control; siHKDC1, knock down HKDC1. * p < 0.05; **p < 0.01; ***p < 0.001; ****p < 0.0001.

**Figure 7:**
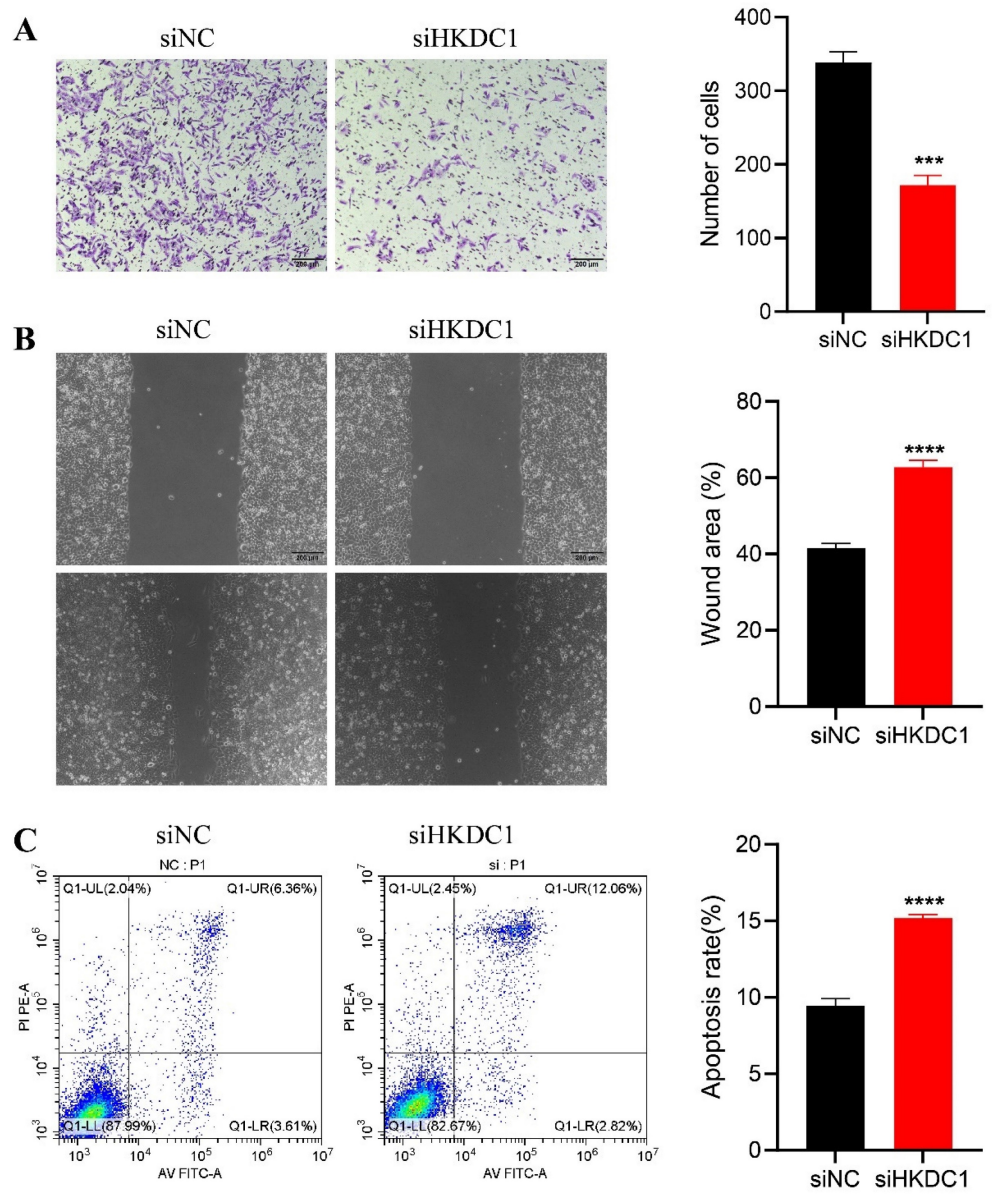
HKDC1 promoted the migration and invasion of PAAD cells and inhibited apoptosis. (A) Transwell assay was used to evaluate the migration and invasion capacity of HKDC1-expressing SW1990 cells. (C, D) The cell migration capacity of SW1990 was evaluated using wound healing experiments. (E) The apoptosis of SW1990 cells was detected by flow cytometry. siNC: knock down interference control; siHKDC1, knock down HKDC1. * p < 0.05; **p < 0.01; ***p < 0.001; ****p < 0.0001.

To reduce the expression of HKDC1, the SW1990 cell line was transfected with siHKDC1, an interfering fragment, and siNC, a control interfering fragment, due to its comparatively elevated expression. qPCR and WB both verified the validity of the transfections (Figure 6A-C). The EDU tests demonstrated that the diminished expression of HKDC1 significantly decreased the proliferative capacity of the pancreatic cancer cells compared to the control group (Figure 6D-E). The colony formation assay demonstrated a significant reduction in the number of colonies in the siHKDC1 group compared to the siNC group (Figure 6F-G). To further examine the impact of HKDC1 on the migratory and invasive capabilities of PAAD cells, a Transwell assay was conducted. The findings indicated that decreased HKDC1 expression had a notable impact on the quantity of cells that migrated and invaded (Figure 7A). Subsequently, the wound healing assay demonstrated a significantly reduced migration rate of siHKDC1 cells compared to control PAAD cells at the 48-hour mark (Figure 7B). In conclusion, flow cytometry analysis demonstrated a notable increase in the apoptosis rate of cells in the siHKDC1 group compared to siNC (Figure 7C).

### HKDC1 promotes glycolysis in PAAD cells

**Figure 8:**
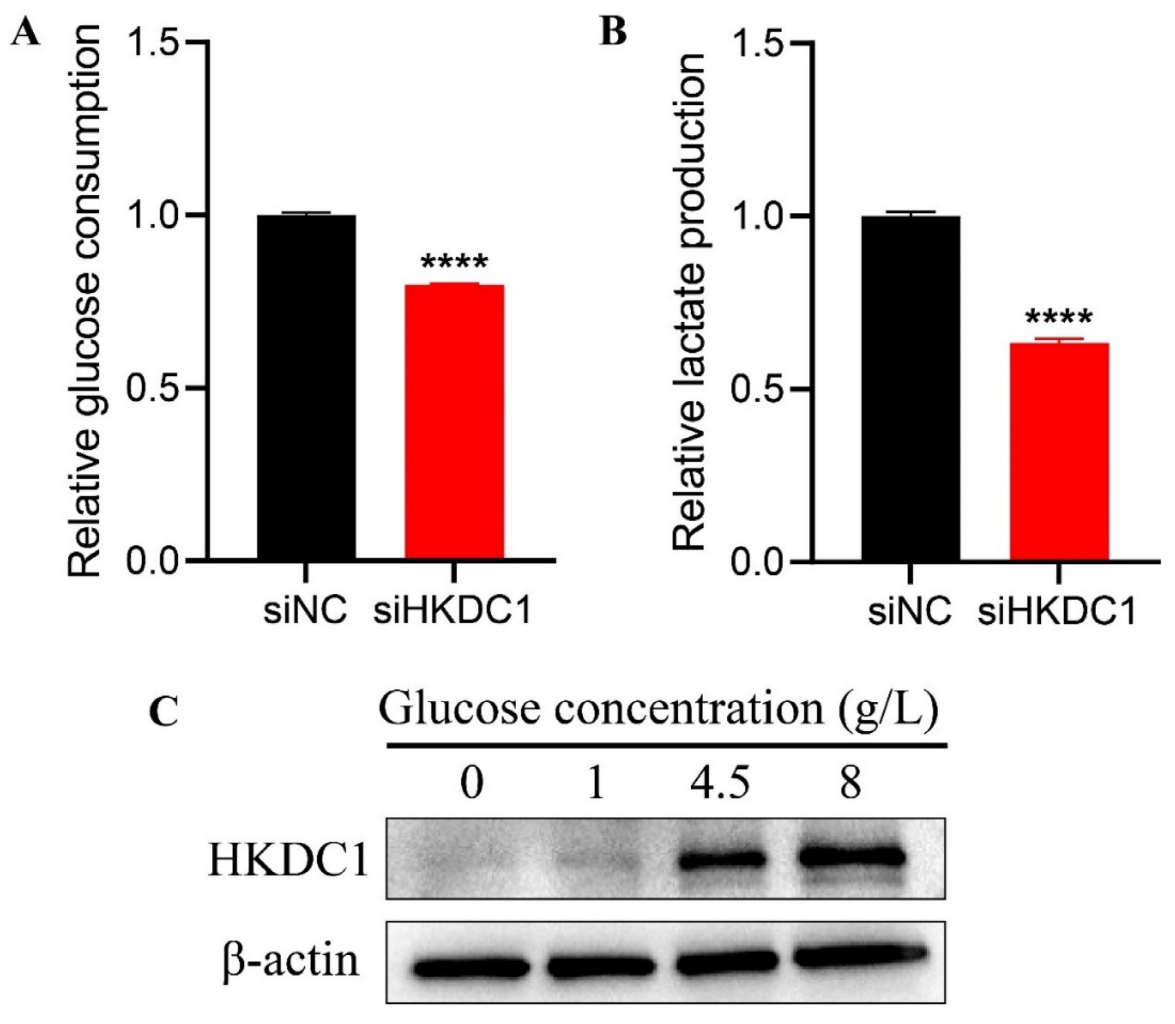
HKDC1 promotes glycolysis and is associated with glucose. (A) Glucose consumption; (B) Lactate production; (C) Different glucose concentrations affected the expression of HKDC1. siNC: knock down interference control; siHKDC1, knock down HKDC1. * p < 0.05; **p < 0.01; ***p < 0.001; ****p < 0.0001.

As HKDC1 functions as a hexokinase, our objective was to investigate the impact of HKDC1 on glycolysis in pancreatic cancer cells. Glucose utilization and lactate generation were observed in cells with low expression of HKDC1 and in control cells, respectively. Figure 8A-B demonstrated that the depletion of HKDC1 resulted in a decrease in glucose utilization and lactate generation in SW1990 cells. Furthermore, altering various glucose concentrations led to the discovery of a range of HKDC1 expression levels (Figure 8C).

## Discussion

This study revealed that HKDC1 exhibited a tendency of elevated expression in PAAD and could potentially function as a standalone prognostic determinant for PAAD. Furthermore, overexpression of HKDC1 can impede the infiltration of immune cells and enhance the proliferation, migration, and glycolysis of PAAD cells, while also suppressing apoptosis.

Previous studies have shown that HKDC1 plays an important role in glucose metabolism and maintains glucose homeostasis 17. Over the past few years, an increasing number of researches have demonstrated that HKDC1 has a tumor-promoting function in various types of malignancies, such as stomach, pulmonary, and hepatic cancers 10-12, 18. The study revealed that PAAD exhibited a notable upregulation of HKDC1, as confirmed through bioinformatics, PCR, and WB assays. Additionally, survival analysis indicated that patients with elevated HKDC1 expression had considerably shorter survival times compared to those with lower HKDC1 expression. Furthermore, our analysis revealed that HKDC1 is a significant prognostic indicator for PAAD based on both unifactorial and multifactorial COX analysis. Based on the amalgamation of prior literature reports and our own discoveries, it can be inferred that HKDC1 has the potential to be a target for treating PAAD and could function as a novel biomarker.

A positive correlation was observed between the expression of HKDC1 and three different types of tumor-initiating cells (TICs), namely resting memory CD4 T cells, M0 macrophages, and M1 macrophages. Conversely, a negative correlation was found with CD8 T cells. The significant roles of CD4 and CD8 T cells in inhibiting tumorigenesis and growth are widely recognized 19, 20. This demonstrates the coherence between our research and the inverse relationship between HKDC1 and CD8 T cells. The elevated level of HKDC1 in pancreatic cancer is associated with reduced CD8 T cells, which could contribute to the poorer prognosis observed in patients with high HKDC1 expression. Furthermore, we assessed the tumor microenvironment using the ESTIMATE algorithm and observed that the stromal score, immune score, and ESTIMATEScore were comparatively reduced in the HKDC1 high-expression group compared to the HKDC1 low-expression group. Besides solid tumors, the tumor microenvironment also contains immune cells and stromal cells. Tumor development relies heavily on the participation of immune cells and stromal cells, making their assessment crucial for diagnosing and predicting the outcome of tumors. Patients with elevated levels of HKDC1 exhibited reduced immune scores and stromal scores, indicating that HKDC1 overexpression hampers the infiltration of immune cells in pancreatic cancer, thereby impeding its anti-tumor effects. Furthermore, patients with high HKDC1 expression displayed a downward trend in ESTIMATEScore, suggesting that their stromal scores were lower compared to those with low HKDC1 expression. Furthermore, the ESTIMATEScore of patients exhibiting elevated HKDC1 levels demonstrated a downward inclination, suggesting that patients with heightened HKDC1 expression may possess increased tumor purity, consequently leading to a heightened level of malignancy. As a result, we postulated that elevated HKDC1 levels could potentially diminish the infiltration of immune cells and stroma, consequently impairing the functionality of immune cells.

Immune Checkpoints, which are a category of molecules that suppress the immune system, have a significant function in controlling immune activation and autoimmunity. The excessive expression of immune checkpoint molecules results in the suppression of immune function, leading to low immunity. Conversely, the insufficient expression of immune checkpoints also causes abnormalities in immune function. During our investigation, we observed a positive correlation between the expression of HKDC1 and immune checkpoints such as LGALS9, TNFRSF14, TNFSF15, CD40, TNFSF9, VTCN1, HHLA2, CD44, and CD276. The findings indicate that the elevated levels of HKDC1 coincide with increased expression of immune checkpoints, resulting in a decline in the body's immune capacity and hindering the antigen presentation process in tumor immunity. Consequently, this leads to the progression of cellular immune evasion in pancreatic cancer. These results support previous findings indicating that increased HKDC1 expression leads to decreased infiltration of immune cells. Furthermore, we assessed the IPS of individuals in various groups expressing HKDC1 using the PAAD-IPS cohort. There was no correlation between IPS values and HKDC1 expression in PAAD patients who received treatment with anti-PD-1 and anti-CTLA4. The findings indicate that individuals with elevated HKDC1 levels are at a greater risk of developing immune resistance or immune evasion. Hence, it may not be advisable to use immunotherapy as the preferred treatment for patients exhibiting elevated levels of HKDC1 expression, as this observation offers valuable information for clinical management. However, a deeper understanding is needed for more in-depth research and exploration.

Furthermore, according to the aforementioned bioinformatics findings, we conducted additional experimental validation to confirm the involvement of HKDC1 in PAAD cells. The findings from our investigation demonstrated that suppressing HKDC1 expression hindered the growth and movement capabilities of PAAD cells while enhancing apoptosis. After suppressing HKDC1 expression, we observed a substantial decrease in both glucose consumption and lactate production, indicating altered cellular response to glucose consumption and lactate production. Notably, we also established through WB analysis that HKDC1 exhibited a strong correlation with glucose levels, with the expression of HKDC1 escalating in tandem with rising glucose concentrations. The findings indicate that HKDC1 plays a part in enhancing the proliferation of PAAD cells and potentially facilitates glycolysis.

To summarize, our investigation revealed a notable increase in HKDC1 expression in PAAD, which was associated with a decreased survival rate among patients. Furthermore, our research discovered that individuals exhibiting elevated levels of HKDC1 expression had a higher propensity for developing immune resistance or immune evasion. In the end, the experiments demonstrated that suppressing HKDC1 resulted in decreased proliferation, diminished movement and infiltration, and enhanced programmed cell death in PAAD cells. The results indicate that targeting HKDC1 could potentially be a treatment option for PAAD and offer novel perspectives on managing the disease.

## Supplementary Material

Supplementary figures and data.

## Abbreviations

PAAD: Pancreatic Adenocarcinoma
HKDC1: Hexokinase Domain Containing 1
HK: Hexokinase
TME: Tumor Microenvironment
OS: Overall Survival
TMB: Tumor Burden
IPS: Immunophenol Scores
EdU: 5-ethynyl-2'-deoxyuridine
TICs: Tumor-Initiating Cells

## References

[1] Klein AP (2021). Pancreatic cancer epidemiology: understanding the role of lifestyle and inherited risk factors. Nature reviews Gastroenterology & hepatology.

[2] Dong L, Lu D, Chen R, Lin Y, Zhu H, Zhang Z (2022). Proteogenomic characterization identifies clinically relevant subgroups of intrahepatic cholangiocarcinoma. Cancer cell.

[3] Guo C, Ludvik AE, Arlotto ME, Hayes MG, Armstrong LL, Scholtens DM (2015). Coordinated regulatory variation associated with gestational hyperglycaemia regulates expression of the novel hexokinase HKDC1. Nature communications.

[4] Hayes MG, Urbanek M, Hivert MF, Armstrong LL, Morrison J, Guo C (2013). Identification of HKDC1 and BACE2 as genes influencing glycemic traits during pregnancy through genome-wide association studies. Diabetes.

[5] Khan MW, Ding X, Cotler SJ, Clarke M, Layden BT (2018). Studies on the Tissue Localization of HKDC1, a Putative Novel Fifth Hexokinase, in Humans. The journal of histochemistry and cytochemistry: official journal of the Histochemistry Society.

[6] Chen QT, Zhang ZY, Huang QL, Chen HZ, Hong WB, Lin T (2022). HK1 from hepatic stellate cell-derived extracellular vesicles promotes progression of hepatocellular carcinoma. Nature metabolism.

[7] Wu S, Zhang H, Gao C, Chen J, Li H, Meng Z (2022). Hyperglycemia Enhances Immunosuppression and Aerobic Glycolysis of Pancreatic Cancer Through Upregulating Bmi1-UPF1-HK2 Pathway. Cellular and molecular gastroenterology and hepatology.

[8] Tuo Z, Zheng X, Zong Y, Li J, Zou C, Lv Y (2020). HK3 is correlated with immune infiltrates and predicts response to immunotherapy in non-small cell lung cancer. Clinical and translational medicine.

[9] Ozaki K, Harada K, Terayama N, Matsui O, Saitoh S, Tomimaru Y (2016). Hepatocyte nuclear factor 1α-inactivated hepatocellular adenomas exhibit high (18)F-fludeoxyglucose uptake associated with glucose-6-phosphate transporter inactivation. The British journal of radiology.

[10] Zhao P, Yuan F, Xu L, Jin Z, Liu Y, Su J (2023). HKDC1 reprograms lipid metabolism to enhance gastric cancer metastasis and cisplatin resistance via forming a ribonucleoprotein complex. Cancer letters.

[11] Wang X, Shi B, Zhao Y, Lu Q, Fei X, Lu C (2020). HKDC1 promotes the tumorigenesis and glycolysis in lung adenocarcinoma via regulating AMPK/mTOR signaling pathway. Cancer cell international.

[12] Khan MW, Terry AR, Priyadarshini M, Ilievski V, Farooq Z, Guzman G (2022). The hexokinase "HKDC1" interaction with the mitochondria is essential for liver cancer progression. Cell death & disease.

[13] Blum A, Wang P, Zenklusen JC (2018). SnapShot: TCGA-Analyzed Tumors. Cell.

[14] Yoshihara K, Shahmoradgoli M, Martínez E, Vegesna R, Kim H, Torres-Garcia W (2013). Inferring tumour purity and stromal and immune cell admixture from expression data. Nature communications.

[15] Newman AM, Steen CB, Liu CL, Gentles AJ, Chaudhuri AA, Scherer F (2019). Determining cell type abundance and expression from bulk tissues with digital cytometry. Nature biotechnology.

[16] Kirby J, Prior F, Petrick N, Hadjiski L, Farahani K, Drukker K (2020). Introduction to special issue on datasets hosted in The Cancer Imaging Archive (TCIA). Medical physics.

[17] Ludvik AE, Pusec CM, Priyadarshini M, Angueira AR, Guo C, Lo A (2016). HKDC1 Is a Novel Hexokinase Involved in Whole-Body Glucose Use. Endocrinology.

[18] Wang MQ, Chen YR, Xu HW, Zhan JR, Suo DQ, Wang JJ (2023). HKDC1 upregulation promotes glycolysis and disease progression, and confers chemoresistance onto gastric cancer. Cancer science.

[19] Knocke S, Fleischmann-Mundt B, Saborowski M, Manns MP, Kühnel F, Wirth TC (2016). Tailored Tumor Immunogenicity Reveals Regulation of CD4 and CD8 T Cell Responses against Cancer. Cell reports.

[20] Ostroumov D, Fekete-Drimusz N, Saborowski M, Kühnel F, Woller N (2018). CD4 and CD8 T lymphocyte interplay in controlling tumor growth. Cellular and molecular life sciences: CMLS.

