# Supplementary figures and images for: HKDC1 enhances the proliferation, migration and glycolysis of pancreatic adenocarcinoma and is linked to immune infiltration
Source: J Cancer. 2024 Feb 12;15(7):1983–93. doi: 10.7150/jca.92823 (PMC10905392; doi:10.7150/jca.92823)

Age 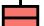 <=65 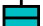 >65

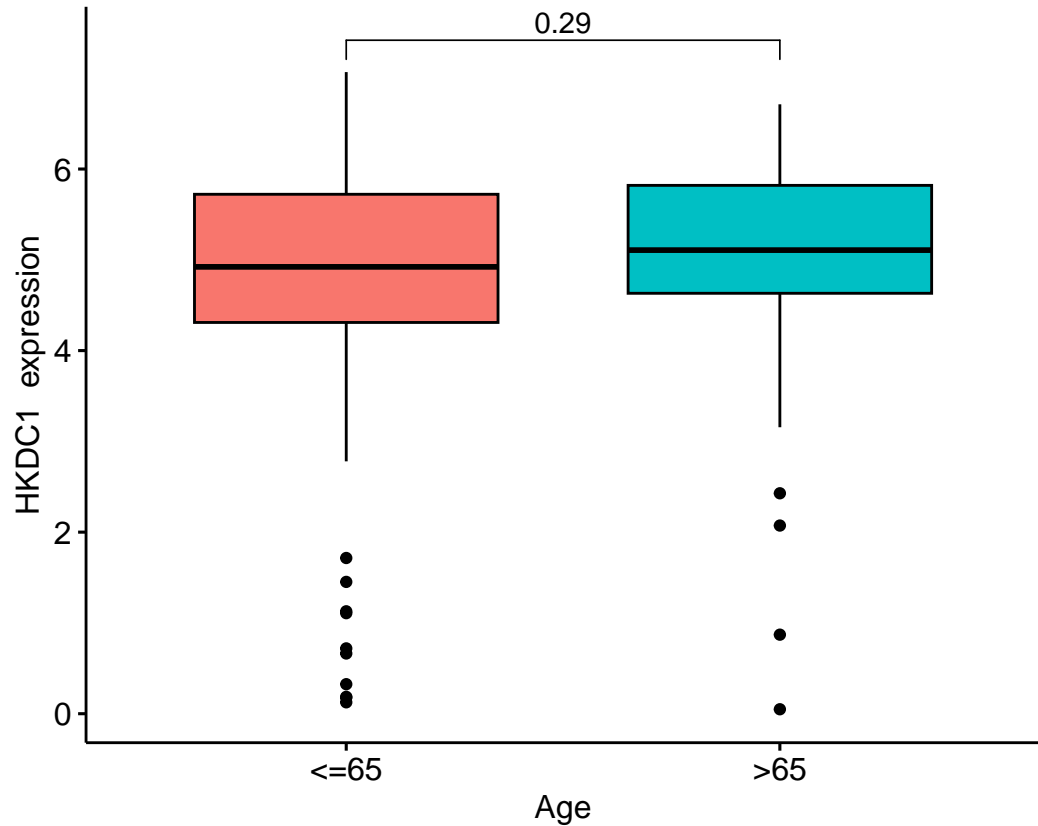

Supplement: Supplementary file 1 — Supplementary figures and data. [file jcav15p1983s1.zip › Supplementary figures and EXCEL/new_clinicalCor_Age.pdf]

Gender 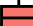 FEMALE 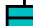 MALE

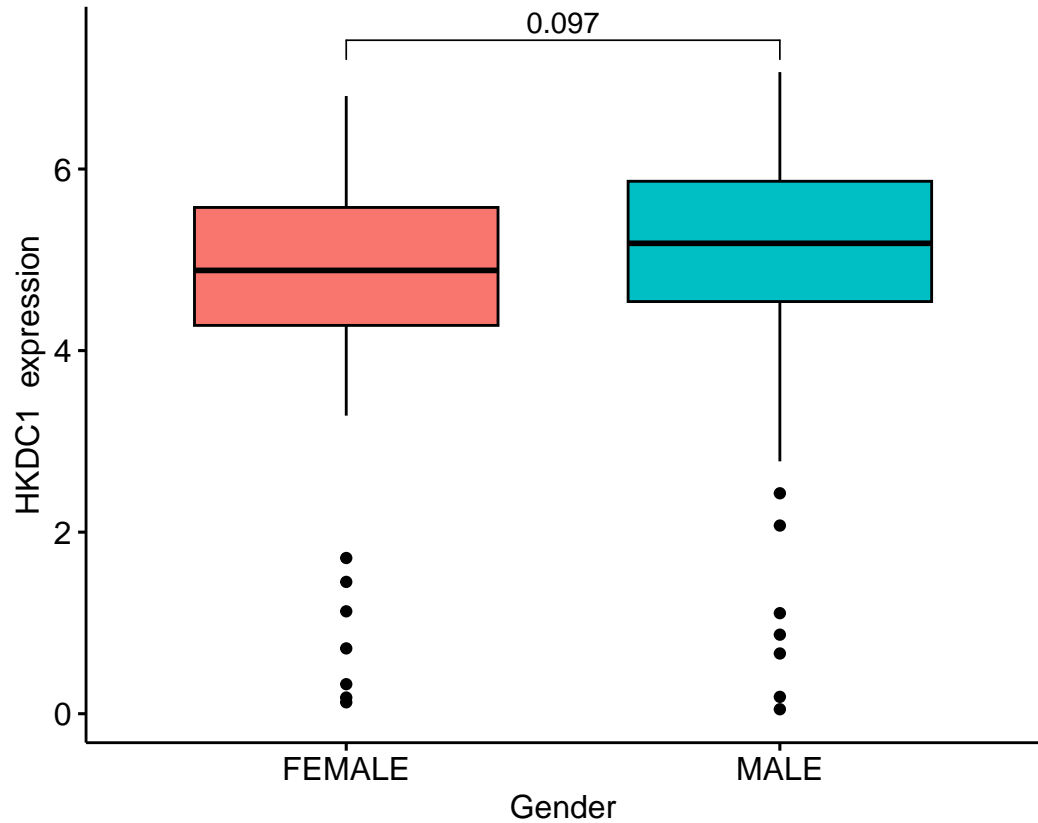

Supplement: Supplementary file 1 — Supplementary figures and data. [file jcav15p1983s1.zip › Supplementary figures and EXCEL/new_clinicalCor_Gender.pdf]

Grade 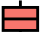 G1-2 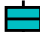 G3-4

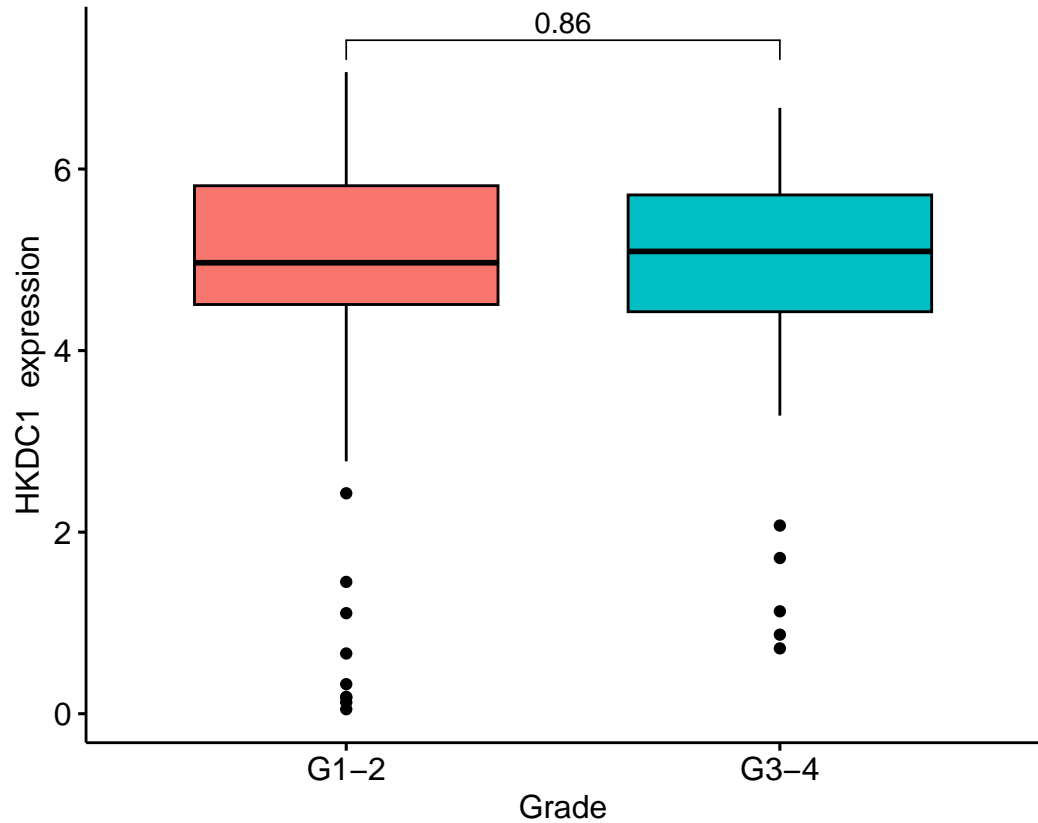

Supplement: Supplementary file 1 — Supplementary figures and data. [file jcav15p1983s1.zip › Supplementary figures and EXCEL/new_clinicalCor_Grade.pdf]

M M0 M1

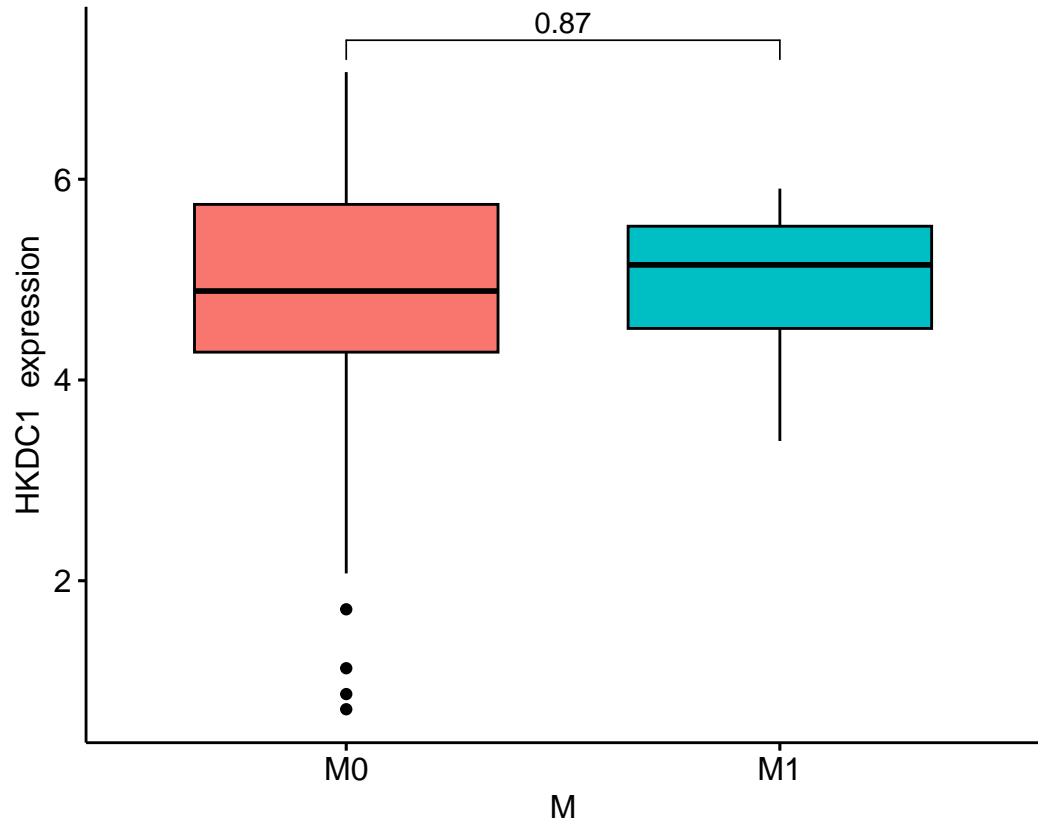

Supplement: Supplementary file 1 — Supplementary figures and data. [file jcav15p1983s1.zip › Supplementary figures and EXCEL/new_clinicalCor_M.pdf]

N 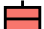 N0 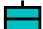 N1

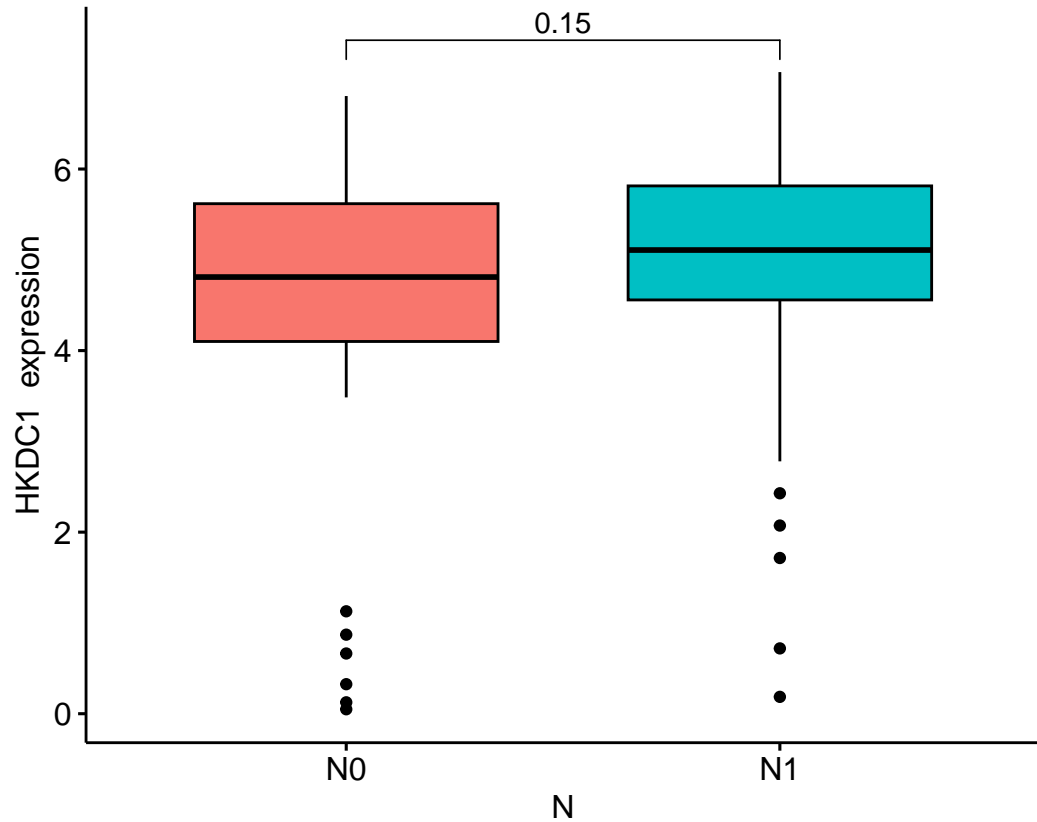

Supplement: Supplementary file 1 — Supplementary figures and data. [file jcav15p1983s1.zip › Supplementary figures and EXCEL/new_clinicalCor_N.pdf]

Stage    Stage I-II    Stage III-IV

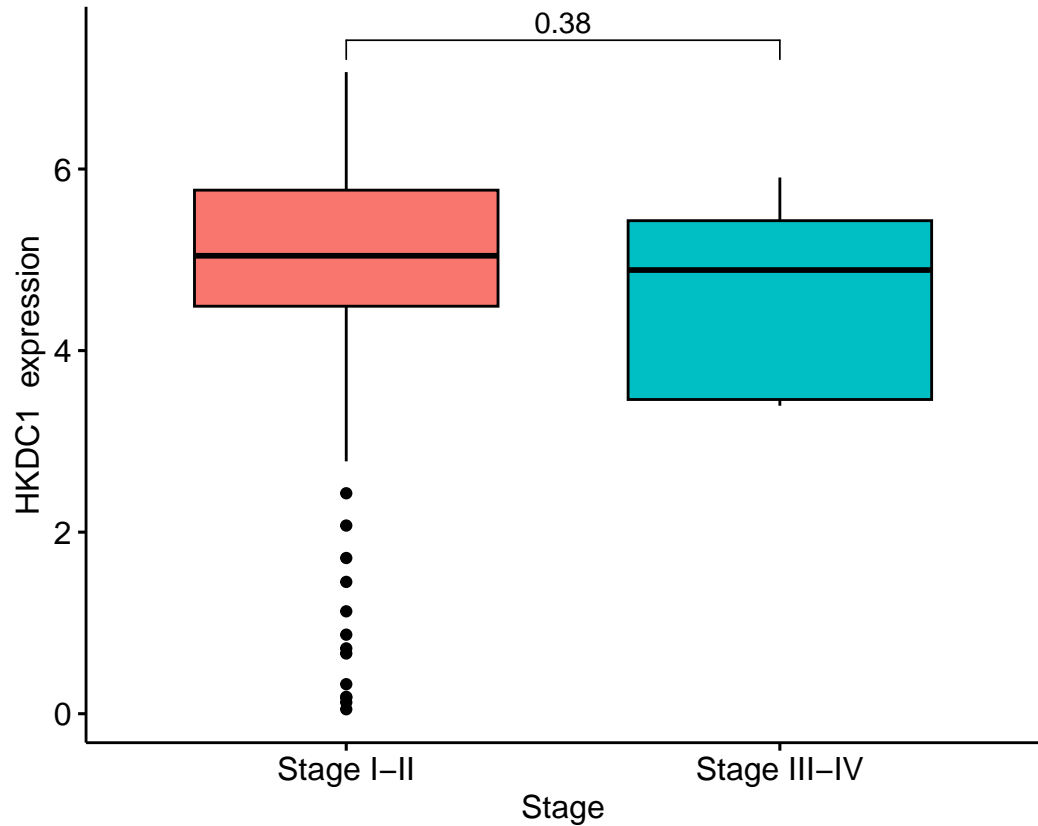

Supplement: Supplementary file 1 — Supplementary figures and data. [file jcav15p1983s1.zip › Supplementary figures and EXCEL/new_clinicalCor_Stage.pdf]

T T1-2 T3-4

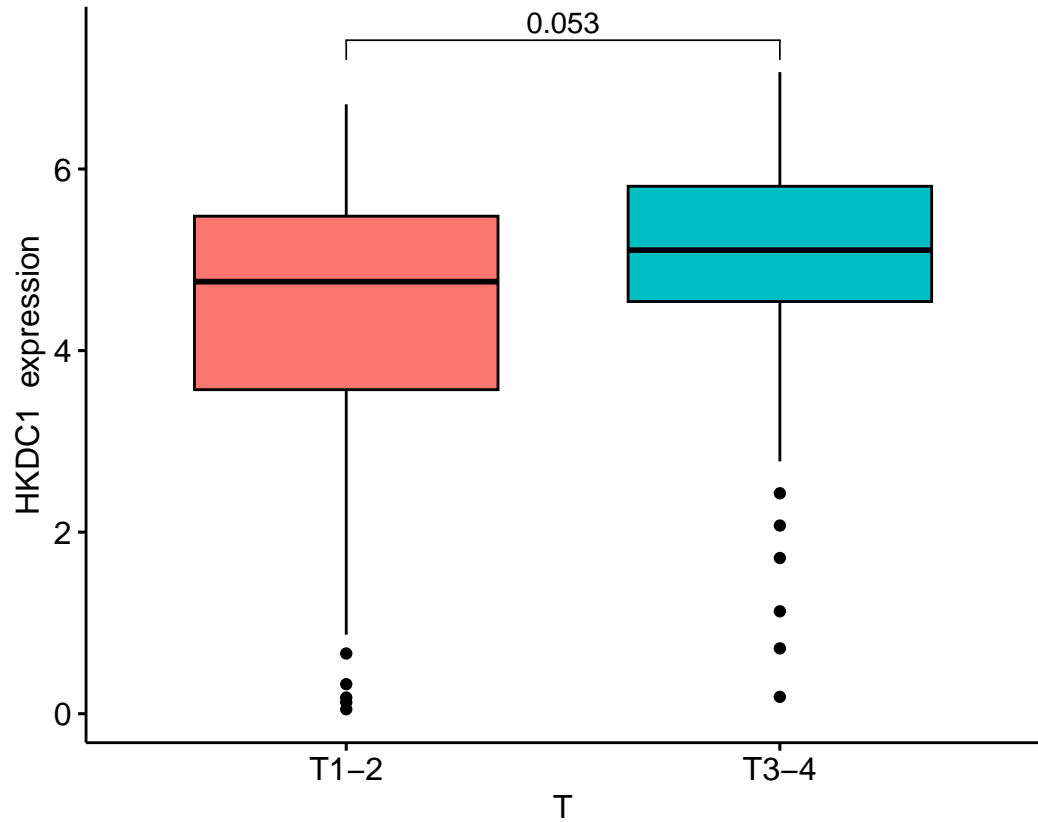

Supplement: Supplementary file 1 — Supplementary figures and data. [file jcav15p1983s1.zip › Supplementary figures and EXCEL/new_clinicalCor_T.pdf]
